# Diseases of the Eye

**Published:** 1904-04

**Authors:** 


### BOOK REVIEWS.

DISEASES OF THE EYE. By L. Webster Fox, A.M., M.D. Prof. Ophthalmology in the Medico-Chirurgical College Philadelphia. Published by D. Appleton & Co., New York and London.

This new work on Ophthalmology by Dr. Fox has much to commend it from the standpoint of the physician and student. As is stated in the preface the arrangement differs from most of the modern works on the same subject, in that "diseases are considered according to the structures attacked in the order in which they are encountered in going from without inward." Dr. Fox has demonstrated by this book that he is a clear, forcible, and practical teacher of ophthalmology, and managed to place before the student many points which too often have been considered as insignificant in importance. While many of the illustrations meet us as old, familiar friends, there are numerous others which are original in character and most excellent in execution. Many of the subjects discussed are illustrated by cases taken from the author's own clinical experience. Each new medical work always shows some points of originality and this one is no exception to the rule. R.

